# Circulating microRNA expression profiling and bioinformatics analysis of dysregulated microRNAs of patients with coronary artery disease

**DOI:** 10.1097/MD.0000000000011428

**Published:** 2018-07-06

**Authors:** Zhixiong Zhong, Jingyuan Hou, Qifeng Zhang, Wei Zhong, Bin Li, Cunren Li, Zhidong Liu, Min Yang, Pingsen Zhao

**Affiliations:** a Center for Cardiovascular Diseases; b Center for Precision Medicine, Meizhou People's Hospital (Huangtang Hospital), Meizhou Hospital Affiliated to Sun Yat-sen University; c Guangdong Provincial Engineering and Technology Research Center for Molecular Diagnostics of Cardiovascular Diseases; d Meizhou Municipal Engineering and Technology Research Center for Molecular Diagnostics of Cardiovascular Diseases; e Clinical Core Laboratory, Meizhou People's Hospital (Huangtang Hospital), Meizhou Hospital Affiliated to Sun Yat-sen University; f Meizhou Municipal Engineering and Technology Research Center for Molecular Diagnostics of Major Genetic Disorders, Meizhou, P.R. China.

**Keywords:** coronary artery disease, microRNAs, RNA sequencing, ROC curves, ST-segment elevation myocardial infarction, unstable angina

## Abstract

MicroRNs (miRNAs) are small non-coding RNAs that modulate the expression of protein-coding genes at the post-transcription level and their dysregulated expression has been implicated in cardiovascular diseases. Circulating miRNAs have been widely recommended as potential biomarkers for many diseases including coronary artery disease. In this study, the miRNA expression profiles of 6 normal coronary artery (NCA), 12 patients with coronary artery disease including 6 unstable angina (UA) patients and 6 ST-segment elevation myocardial infarction (STEMI) patients were determined by small RNA sequencing. The differential expression of miRNAs was verified via using quantitative reverse transcription polymerase chain reaction (qRT-PCR). We further performed bioinformatics analysis for the differentially expressed miRNAs. The results showed that 60 miRNAs were up-regulated and 26 miRNAs were down-regulated in the UA group and 49 miRNAs were up-regulated and 62 miRNAs were down-regulated in the UA group when compared with the NCA group. Among them, both of UA group and STEMI group shared 38 dysregulated miRNAs (28 up-regulated and 10 down-regulated) versus NCA group. ROC curves analysis showed that miR-142-3p and miR-17-5p might server as potential biomarkers for the detection and diagnosis of UA and STEMI. Bioinformatics functional predictions showed that the differential expressed miRNAs were closely related with the pathological process of coronary artery disease. We comprehensively analyzed profile expression of circulating miRNAs of patients with coronary artery disease. Our study suggested that miR-142-3p and miR-17-5p might be potential targets for follow-up research in evaluating biomarkers of coronary artery disease.

## 1 Introduction

Despite tremendous advances in the treatment of coronary artery disease (CAD), it remains one of the leading cause of morbidity and mortality around the world, causing a major social and economic burdens.^[1,2]^ Usually, the occurrence and development of CAD results from multistep atherosclerosis, and these involve in various changes of lifestyle and genetic factors.^[3,4]^ To date, a number of epidemiological studies have identified several risk factors for CAD, including dyslipidaemia, diabetes, hypertension, smoking, and dietary.^[5–7]^ Increased evidence has showed that aberrant change in the expression of multiple genes in CAD plays a critical role in the pathogenesis of atherosclerosis.^[8,9]^ However, the precise molecular mechanisms that regulate cardiac gene expression in the initiation and progression of CAD remain unclear.

Over the past few decades, developments in the sequencing technology and bioinformatics have led to the discovery of that almost all of the human genomes are transcribed with a large number of non-coding RNAs (ncRNAs), including microRNAs (miRNAs), long ncRNAs, and circular RNAs.^[10,11]^ miRNAs are a class of endogenous short, single-stranded noncoding RNAs with approximate 22 nucleotide in length and are is relatively stable in cells, tissues, and body fluids including plasma or serum.^[12–14]^ It has been well established that circulating miRNAs modulate the expression of protein-coding genes at the post-transcription level and are involved in a variety of biological functions and processes including inflammation, development, differentiation, apoptosis and innate immunity.^[15–18]^ Dysregulation of miRNAs expression is strongly associated with various diseases, including atherosclerosis, nervous system disorders, and cancer.^[19–21]^ Moreover, emerging data have demonstrated that miRNAs are critically involved in the pathogenesis of CAD.^[22,23]^ The aberrant circulating miRNAs in CAD may not only indicate the pathological state of CAD but also related to clinical progression.^[24]^ Therefore, comprehensive estimations and analyses of the miRNAs underlying the pathogenesis of CAD are essential to develop effective strategies for the diagnosis and treatment of CAD.

In the present study, an RNA sequencing approach was utilized to systematically identify the expression profiles of miRNAs in plasma of unstable angina (UA), ST-segment elevation myocardial infarction (STEMI), and normal coronary artery (NCA). Finally, potential functions of the deregulated miRNA by gene ontology (GO) and Kyoto Encyclopedia of Genes and Genomes (KEGG) enrichment were analyzed as well. To investigate the diagnostic potential of miRNAs for distinguishing UA and STEMI from NCA, ROC curve analysis was also plotted.

## 2 Methods

### 2.1 Study population

All subjects enrolled from Meizhou People's Hospital (Huangtang Hospital), Meizhou Hospital Affiliated to Sun Yat-sen University, Guangdong, China between January 2015 and December 2016. All the subjects in this study visited Department of Cardiovascular Diseases of Meizhou People's Hospital (Huangtang Hospital), Meizhou Hospital Affiliated to Sun Yat-sen University, Guangdong, China for reasons of chest pain and angina pectoris. Inclusion criteria: cardiovascular risk factors, symptoms of chest pain, ischemic changes in electrocardiograph (ECG), or elevated myocardial enzymes. Exclusion criteria: impaired left ventricular ejection fraction ≤45%, congestive heart failure, chronic kidney or hepatic disease, acute myocardial infarction, and malignant disease.

All subjects in this study were examined by laboratory tests, ECG, and coronary angiography. According to the examination findings, subjects were divided into 2 groups, that is, normal coronary artery (NCA, n = 26), unstable agina (UA, n = 26), and ST-segment elevation myocardial infarction (STEMI, n = 26). In UA group patients had a history of angina (within 1 month), irregular angina at rest or with minimal exertion, and no elevation in troponin. For the STEMI group, only patients diagnosed with a new-onset STEMI based on the admission of ST-segment changes on ECG and elevated serial troponin levels were included in this study. A significant atherosclerotic lesion is defined as at least 50% stenosis in any of the major coronary arteries or in the left main trunk and the diagnosis was assessed by coronary angiography. Coronary angiographic results were confirmed by at least 2 senior cardiologists according to the European Society of Cardiology/American College of Cardiology. Clinical history and medication records were collected. The study was performed under the guidance of the Helsinki Declaration and approved by the ethical committee of Meizhou People's Hospital (Huangtang Hospital), Meizhou Hospital Affiliated to Sun Yat-sen University, Guangdong, China. Informed consent was obtained from all patients and was approved by all centers.

### 2.2 Sample collection and RNA extraction

Five milliliters of venous blood were collected in from each subject in ethylenediaminetetraacetic acid anticoagulant tube, then immediately centrifuged (5810R, Eppendorf, Germany) at 1500 × *g* for 10 minutes at room temperature, followed by a 10 minutes high-speed centrifugation at 12,000 × *g* at 4 °C to completely remove cell debris. The resulting serum was aliquoted in batches of 500 μL and stored at −80 °C until RNA extraction.

Total RNA from 500 μL serum was extracted using the miRNeasy Mini kit (Qiagen, Hilden, Germany) according to the manufacturer's protocol. Total RNA concentration was determined using Qubit RNA assay kit in Qubit 2.0 Flurometer (Life Technologies, CA) and RNA integrity was assessed using the RNA Nano 6000 Assay Kit of the Agilent Bioanalyzer 2100 system (Agilent Technologies, CA).

### 2.3 miRNA validation by qRT-PCR

Quantitative reverse transcription polymerase chain reaction (qRT-PCR) was performed to assess the reproducibility and reliability of the differentially expressed miRNAs identified by small RNA sequencing analysis. Total RNA (1 μg) was reverse transcribed (RT) to cDNA using Bulge-Loop miRNA qRT-PCR Starter Kit (RiboBio Co., Ltd., China) in RT reaction (42 °C, 1 hour, 70 °C, 10 minutes, 4 °C∞), according to the manufacturer's instructions. The cDNA was amplified using miRNAs assay primers and the SYBR Green Mix (RiboBio Co., Ltd., China) according to the manufacturer's instructions on the Roche Lightcycler 480. The primer sequences are presented in Table 1. In addition, a common reverse primer was used in this experiment as follow: 5′-CAGTGCGTGTCGTGGAGT-3′. Each reaction was performed under the following conditions: initialization for 10 minutes at 95 °C, and then 40 cycles of amplification, with 2 seconds at 95 °C for denaturation, 20 seconds at 60 °C for annealing, and 10 seconds at 70 °C for elongation. All cDNA samples were quantified in triplicate and mean cycle threshold (Ct) was calculated from the duplicate PCRs. In addition, we included a no-template control and no-reverse transcriptase control in each run. The relative miRNAs expression levels were normalized to U6 snRNA expression and the fold change between 2 groups was calculated using the 2^-ΔΔCt^ method.

**Table 1:**
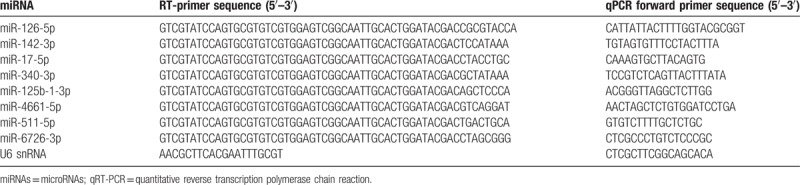
Primers used for qRT-PCR.

### 2.4 Small RNA sequencing and data process

A total amount of 3 μg total RNA per sample was used as input material for the small RNA library. Sequencing libraries were generated using NEBNext Multiplex Small RNA Library Prep Set for Illumina (NEB, MA) following manufacturer's instructions. Briefly, the small RNAs were ligated with RNA adapter followed by reverse transcription using RT primers. Following PCR amplification of the adaptor enriched fragments, the PCR-amplified cDNAs were purified on a 8% polyacrylamide gel to obtain the small RNA fragments corresponding to 140–160 bp. MicroRNA sequencing was performed on an Illumina Hiseq 2500 platform (Illumina Inc., CA) and 50 bp single-end reads were generated.

Raw reads of fastq format were firstly processed through custom perl and python scripts. After masking of the adaptor sequences and removal of contaminated reads, clean reads were obtained. At the same time, Q20, Q30, and GC-content of the raw reads were calculated. Then, an appropriate range of length from clean reads was chose to do all the downstream analyses. Raw sequencing data were mapped to the human genome sequence by Bowtie. Mapped small RNA tags were used to looking for known miRNA by using a reference miRNAs database from miRBase v20.0. Identification of the novel miRNAs was performed by using miREvo (http://evolution.sysu.edu.cn/software/mirevo.htm, Guangzhou, China) and mirdeep2 software (Biomarker Technologies, Beijing, China). Hierarchical cluster, a method of unsupervised cluster analysis, was used to achieve the clustering of the different miRNAs and groups of samples, enabling the detection of the differential effects of miRNA in the different groups. Differentially expressed miRNAs of 2 groups was performed using the DEGseq R package. A cutoff criteria of log2 fold change (FC) >1 and *P* < .05 was considered to be significantly differentially expressed.

### 2.5 Function and pathway enrichment analysis

Function and pathway enrichment analysis of the identified target genes of differentially expressed miRNAs were performed. The functional enrichment analysis was conducted using gene ontology (GO) consisted of biological processes, cellular components, and molecular functions terms via using the software Database for Annotation Visualization and Integrated Discovery (DAVID) (https://david.ncifcrf.gov/, MD). Pathway analysis was based on the Kyoto Encyclopedia of Genes and Genomes (KEGG) database that determine the pathways affected by the target genes with differential miRNA expression. In detailed, the enriched biological processes, cellular components, molecular functions, and signaling pathways were sorted out through hyper-geometric and Fisher tests after mapping the potential target genes to the dataset of GO annotations and KEGG pathways. False discovery rate (FDR) adjustment was performed to judge the significance of differences in multiple testing and a *q* < 0.05 was considered to indicate a statistically significant result.

### 2.6 Statistical analyses

All statistical analyses were performed using the SPSS statistical package, version 19.0 (SPSS Inc., Chicago, IL). Continuous variables were expressed as mean values with standard deviations (SD). Categorical data were expressed as counts and percentages. Student *t* test was used to compare the differences between 2 groups. The one-way analysis of variance (ANOVA) was used to determine the differences among the 3 groups. A receiver operating characteristic^[19]^ curve was generated for each miRNA. Areas under the curve (AUC) and their respective 95% confidence intervals (CI) were calculated to evaluate the sensitivity and specificity for predicting CAD. A *P* value of <.05 was considered statistically significant.

## 3 Results

### 3.1 Patient characteristics

To assess the plasma miRNA expression profiles in CAD, a total of 6 UA patients, 6 STEMI patients, and 6 NCA were recruited for the small RNA sequencing analysis in this study. The clinical characteristics of the enrolled patients according to groups are summarized in Table 2. The mean age and male to female ratio were similar between the 3 groups. There was no significant difference in the distribution of smoking, drinking, hypertension, diabetes mellitus, dyslipidemia, high density lipoprotein cholesterol (HDL-C), and low density lipoprotein cholesterol (LDL-C) of patients among groups. However, the total cholesterol (TC) and triglycerides it is significantly increased in UA group and STEMI group compared with NCA group (*P* < .05). A total of 20 UA patients, 20 STEMI patients, and 20 NCA subjects were also studied for the qRT-PCR validation. The baseline clinical characteristics of the study subjects are shown in Table 2. There no statistical differences were observed excepted TC and LDL-C (*P* < .01) among the 3 groups.

**Table 2:**
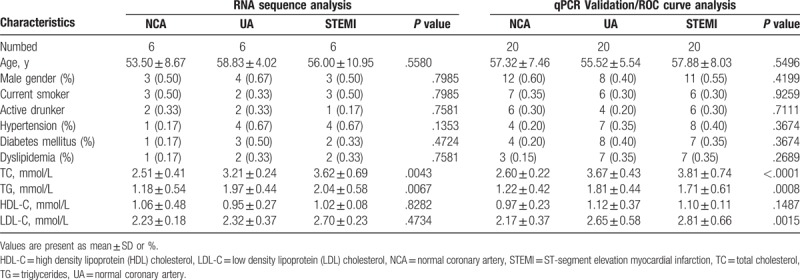
Clinical characteristics of patients enrolled in our study.

### 3.2 Screening of differentially expressed miRNAs profiles

**Figure 1:**
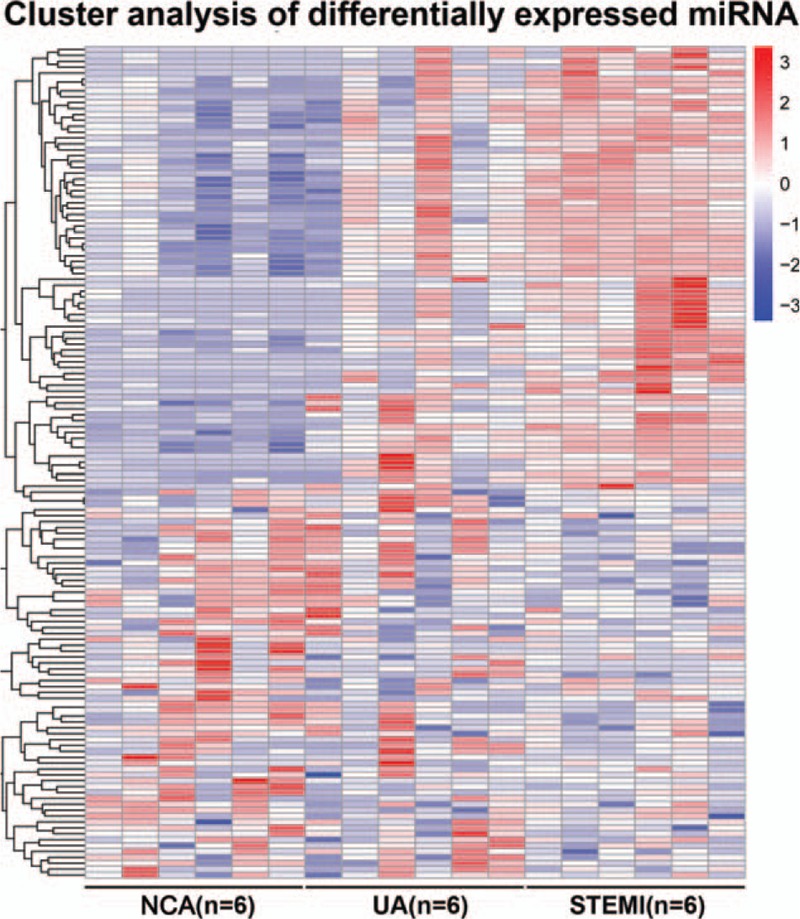
Hierarchical clustering heat map of the differentially expressed miRNAs in the 3 study groups (UA, STEMI, and NCA). The red shade areas represent relatively high miRNAs expression, whereas the blue shade areas represent relatively low miRNAs expression. The miRNAs in the heat map are clustered based on the relative expression patterns. miRNAs = microRNAs; NCA = normal coronary artery; STEMI = ST-segment elevation myocardial infarction; UA = unstable angina.

We determined the plasma miRNA expression profiles in 6 UA patients, 6 STEMI patients, and 6 NCA group using the small RNA sequencing analysis. To identify groups of samples with specific patterns of miRNAs’ expression, we performed unsupervised hierarchical cluster analysis with complete method on all miRNAs which allowed us to investigate similarities or differences between the CAD and NCA samples. A heat map of the hierarchical clustering was generated and the results revealed a clearly distinct expression of all differentially expressed miRNAs in patients with UA and STEMI compared with NCA group, as shown in Fig. 1. With the cutoff criteria of log2 (fold-change) >1 and *P* < .05, 60 miRNAs were up-regulated and 26 miRNAs were down-regulated in the UA group and 49 miRNAs were up-regulated and 62 miRNAs were down-regulated in the STEMI group when compared with the NCA group. Specifically, both of UA group and STEMI group shared 38 dysregulated miRNAs (28 up-regulated and 10 down-regulated) versus NCA group, as summarized in Table 3. None of the 3 miRNAs differed significantly between UA and STEMI patients.

**Table 3:**
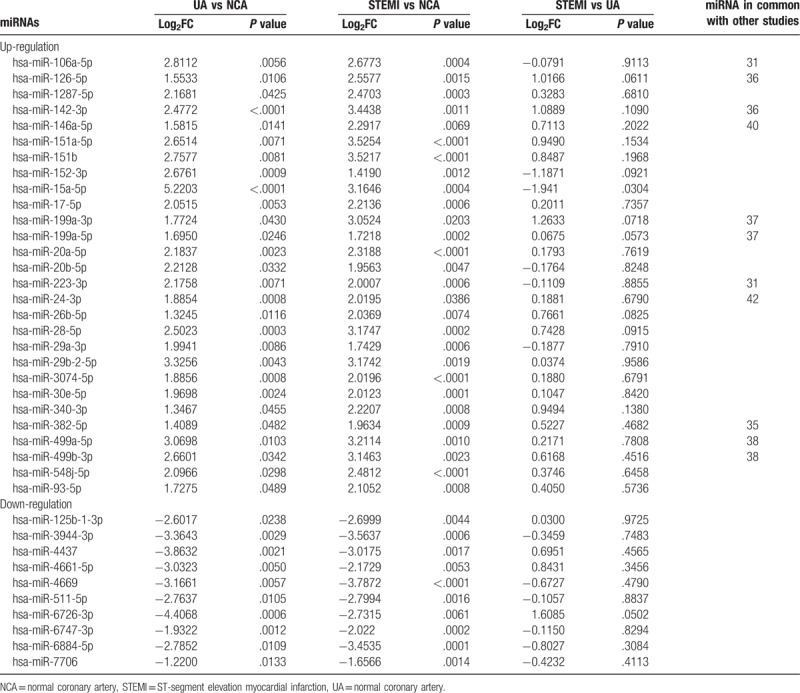
List of miRNAs found differentially expressed between NCA, UA, and STEMI.

### 3.3 Validation of the RNA sequencing results by qRT-PCR

**Figure 2:**
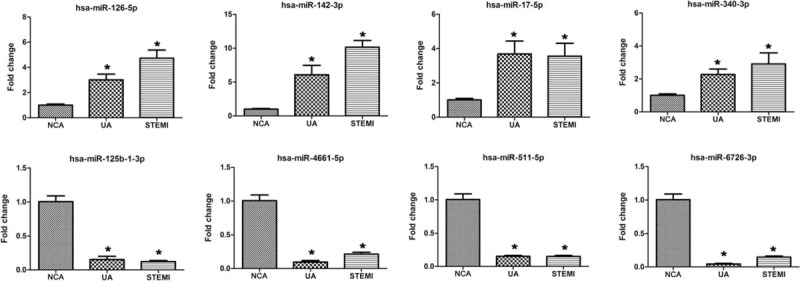
qPCR validations of 8 dysregulated miRNAs in the sample of NCA, UA, and STEMI. The 8 candidate miRNAs were evaluated with qRT-PCR using 60 samples, including 20 NCA, 20 UA, 20 STEMI samples. ∗*P* < .05. miRNAs = microRNAs; NCA = normal coronary artery; qRT-PCR = quantitative reverse transcription polymerase chain reaction; STEMI = ST-segment elevation myocardial infarction; UA = unstable angina.

In an attempt to validate the reliability and robustness of the sequencing results, 8 miRNAs with abnormal expression, namely, miR-126-5p, miR-142-3p, miR-17-5p, miR-340-3p, miR-125b-1-3p, miR-4661-5p, miR-511-5p, and miR-6726-3p were randomly selected and analyzed by qRT-PCR in plasma samples of 20 patients with UA, 20 patients with STEMI, and 20 NCA. To ensure that no biases were present in the qRT-PCR data, blank control without template and reverse transcription were also included to evaluate PCR amplification of contaminating genomic DNA and the amplification result was negative. Consistent with the small RNA sequencing data, the expression of these miRNAs was either up-regulated or down-regulated in the UA group and STEMI group in comparison with the NCA group (Fig. 2). All the selected miRNAs have shown a consistent result in qRT-PCR with sequencing data using RNU6B as an endogenous control.

### 3.4 Diagnostic potential of miRNAs

**Figure 3:**
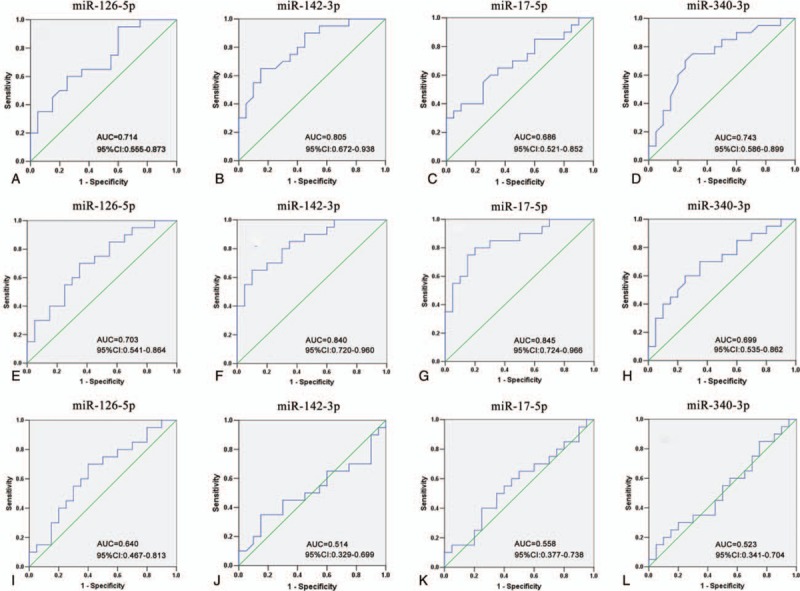
The receiver operating characteristic^[19]^ curve were analysis for discriminative ability among NCA, UA, and STEMI by the 4 upregulated miRNAs. UA versus NCA (A–D); STEMI versus NCA (E–H)^[1]^; STEMI versus NCA (I–L). 95% CI = 95% confidence interval; AUC = area under the ROC curve; miRNAs = microRNAs; NCA = normal coronary artery; qRT-PCR = quantitative reverse transcription polymerase chain reaction; STEMI = ST-segment elevation myocardial infarction; UA = unstable angina.

**Figure 4:**
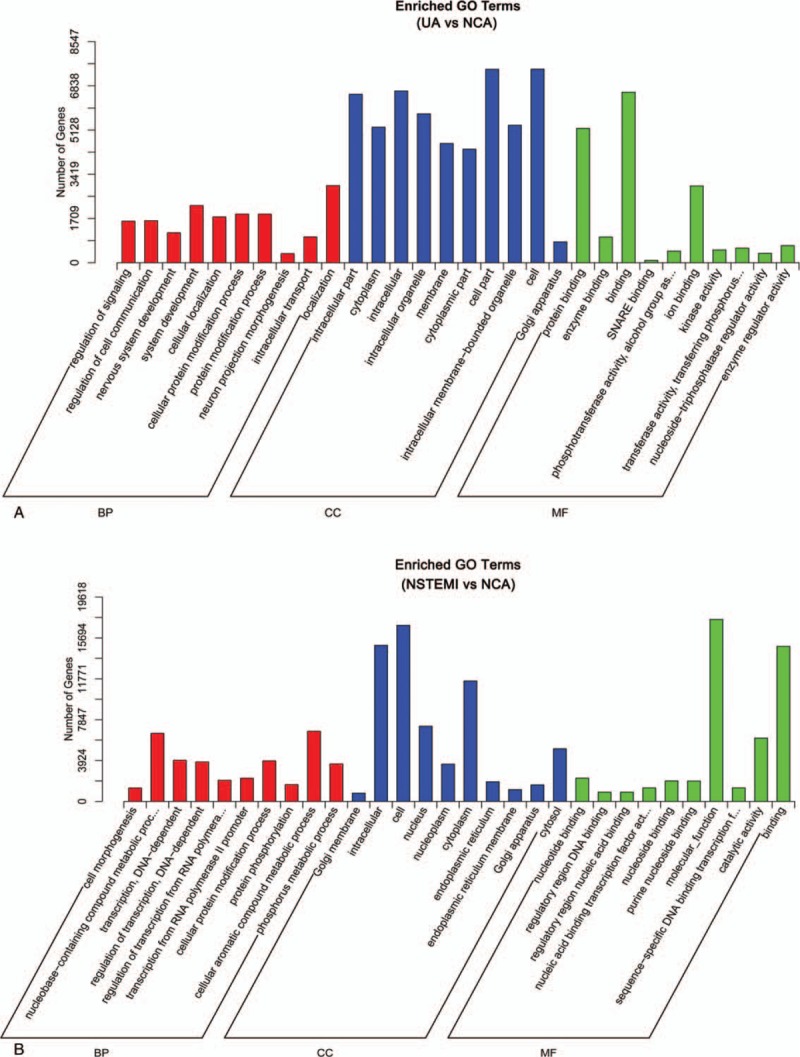
Gene ontology enrichment analysis of target genes of differently expressed miRNAs. (A) UA versus NCA; (B) STEMI versus NCA. miRNAs = microRNAs; NCA = normal coronary artery; STEMI = ST-segment elevation myocardial infarction; UA = unstable angina.

To investigate the diagnostic potential of miRNAs for distinguishing UA and STEMI from NCA, ROC curve analysis was also plotted. Considering the feasibility and convenience to be a diagnostic marker, 4 significantly up-regulated miRNAs in UA patients and STEMI patients validated above were selected, namely, miR-126-5p, miR-142-3p, miR-17-5p, miR-340-3p. For UA patients, the ROC curves of miR-142-3p showed a relatively higher distinguishing efficiency from NCA with an AUC value of 0.805 (95% CI: 0.672–0.938). While the ROC curves of miR-126-5p, miR-17-5p, and miR-340-3p exhibited a moderate distinguishing efficiency with an AUC value of 0.714 (95% CI: 0.555–0.873), 0.686 (95% CI: 0.521–0.852), and 0.743 (95% CI: 0.586–0.899), respectively (Fig. 3A, C, D). For STEMI patients, both miR-142-3p and miR-17-5p showed a higher distinguishing efficiency with an AUC value of 0.840 (95% CI: 0.720–0.960) and 0.845 (95% CI:0.724–0.966) (Fig. 4F and G), while miR-126-5p and miR-340-3p exhibited a lower distinguishing efficiency with an AUC value of 0.703 (95% CI: 0.541–0.864) and 0.699 (95% CI: 0.535–0.862) (Fig. 4E and H), respectively. However, none of these miRNAs reached an acceptable AUC value to differentiate SA from UA patients, ranging from 0.404 (miR-145) to 0.678 (miR-337-5p). Detailed information on the ability of these 4 lncRNAs to differentiate between patients with UA and STEMI is presented in Fig. 4(I–L).

### 3.5 Bioinformatics analysis and functional prediction

**Figure 5:**
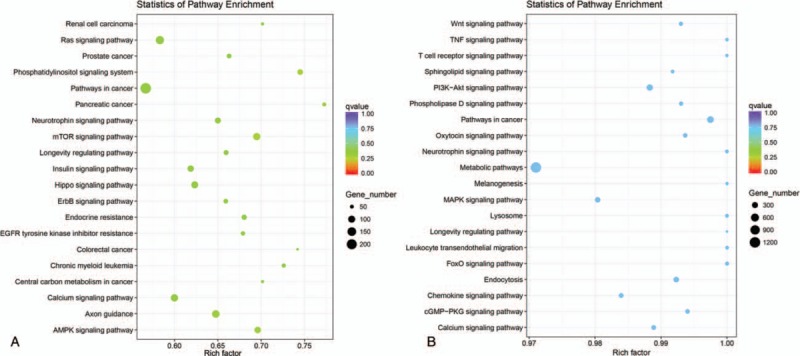
Kyoto encyclopedia of genes and genomes analysis of target genes of differently expressed miRNAs. (A) UA versus NCA; (B) STEMI versus NCA. miRNAs = microRNAs; NCA = normal coronary artery; STEMI = ST-segment elevation myocardial infarction; UA = unstable angina.

A functional enrichment analysis was performed to examine the enrichment of annotated terms of target genes of differentially expressed miRNAs in patients with UA and STEMI compared with NCA. A total of 373,642 and 543,411 total target genes in UA and STEMI group, respectively, were generated, and 20,629 and 20,852 unique genes were identified. After adjusting the number of total genes count annotated by a FDR *q* value <0.05 to obtain the most convincing result for enrichment analysis, the top 10 most enriched GO terms including biological processes, cellular components, and molecular functions were presented in Fig. 4. Among which, GO functional analysis of the target genes of differentially expressed miRNAs in UA group revealed these differentially expressed target genes were markedly involved in several biological processes, including regulation of signaling, regulation of cell communication, cellular localization, and protein modification process. While in STEMI group, the differentially expressed target genes were significantly involved in transcription, nucleobase-containing compound metabolic process, regulation of transcription from RNA polymerase II promoter, protein modification process, and cellular aromatic compound metabolic processes (Fig. 4A and B). Pathway significance concentration analysis revealed that the differentially expressed target genes were involved in various pathways, including Ras signaling pathway, mammalian target of rapamycin (mTOR) signaling pathway, insulin signaling pathway, hippo signaling pathway, and calcium signaling pathway in UA group (Fig. 5A). Moreover, differentially expressed miRNAs targets in STEMI group compared with that in NCA were found to regulate biological processes such as PI3K-Akt signaling pathway, metabolic pathways, MAPK (mitogen-activated protein kinase) signaling pathway, and calcium signaling pathway (Fig. 5B). Bioinformatics functional predictions showed that the differential expressed miRNAs might be association the pathological process of cardiovascular disease.

## 4 Discussion

CAD remains one of the leading cause of morbidity and mortality and has become a global health problem worldwide.^[2]^ Although tremendous progress has been made in the treatment of CAD with the introduced of percutaneous coronary intervention (PCI) and the application of stents, there still large number of patients suffer from serious cardiovascular events.^[25,26]^ Up till now, the pathogenesis of CAD has not been clearly elucidated but most likely it involves atherosclerosis, as well as environmental and genetic factors.^[3,4]^ In the present study, we performed a small RNA sequencing analysis to assess the circulating miRNAs expression levels of UA patients, STEMI patients, and NCA. Interestingly, we identified 38 miRNAs, whose expression level were consistently changed in UA and adult STEMI patients compared with NCA, demonstrating the dynamic changes with the progression of CAD. Further functional analysis of the selected miRNAs suggests that they might play a critical role by targeting several genes associated with important biological process and pathways during the pathological process of CAD.

Accumulating studies have reported that miRNAs, a group of endogenous small noncoding RNAs, act as critical posttranscriptional regulators of gene expression and their roles have been described widely in physiologic and pathologic processes.^[15,18]^ Recent studies have observed that miRNAs exhibit remarkable biostability when present in the circulation and several reports have highlighted the potential role of the circulation miRNAs in patients with cardiovascular diseases. For instance, a prior study have determined the transcardiac gradient of cardio-microRNAs in failing hearts, suggesting their value as potential biomarkers for heart failure (HF) in the circulation.^[27]^ Likewise, another research suggested that circulating levels of miRNAs are differentially expressed in patients with HF of different etiologies, and might be useful biomarkers to distinguish HF of different etiologies.^[28]^ Circulating levels of vascular and inflammation-associated microRNAs have also been shown significantly downregulated in patients with CAD. Meanwhile, elevated cardiac-specific miR-208a has previously been associated with the myocardial injury both in AMI rats model and patient's plasma.^[29]^ In our study, a clearly distinct expression of all differentially expressed miRNAs in patients with UA and STEMI compared with NCA group and 38 dysregulated miRNAs were shared among NCA, UA, and STEMI (Table 1). Strikingly, many of these miRNAs are involved in various biological processes including angiogenesis, inflammation, proliferation, migration, and apoptosis by targeted mRNAs, as report previously in the literatures.^[30]^ For instance, hsa-miR-199a-3p was shown to promote cardiomyocyte proliferation and, thus, cardiac regeneration and functional recovery in the myocardial infarction mice model.^[21]^ MiR-142-3p negatively regulates augmented IL-6 production in neonatal polymorphonuclear leukocytes.^[31]^ Recent evidence indicated that hsa-miR-146a-5p was a potential molecular target for inhibiting inflammation and apoptosis in the diabetic retina through the suppression of the IL-6-mediated STAT3/VEGF pathway.^[32]^ Recent studies have also demonstrated that RP5-833A20.1/miR-382-5p/NFIA-dependent signal transduction pathway contributes to the regulation of cholesterol homeostasis and inflammatory reaction.^[33]^ Based on these observations, we can hypothesize that deregulated miRNAs may critically affect the progression of CAD. However, the underlying mechanisms remain unclear.

Over the past years, miRNAs expression profiles have become the focus of intensive research in order to reach improved diagnostics of CAD. In the present study, we reported the profile of miRNAs in serum derived from patients with UA and STEMI, as well as NCA. From an initial screening of miRNAs, our results showed that 38 dysregulated miRNAs were present at consistently increased or decreased expression levels both in UA patients and STEMI patients when compared with healthy controls. The results demonstrated that those miRNAs exhibiting a trend of consistent expression were predominantly upregulated. Therefore, we hypothesized their expression may be closely associated with pathological process of CAD, other studies have also reported the similar results with ours. Among these, miRNAs including miR-126-5p, miR-142-3p, miR-15a-5p, miR-17-5p, miR-199a-3p, miR-199a-5p, miR-223-3p, miR-24-3p, miR-26b-5p, miR-28-5p, miR-499a-5p, and miR-499b-3p have the potential effects involved in endothelial cell proliferation migration, inflammation, angiogenesis, neointima formation, and apoptosis in various atherosclerotic disease.^[34–38]^ In addition, an inconsistent trend was also found in patients with UA and STEMI versus healthy controls. For example, miR-140-5p, miR-146b-5p, and miR-21-5p in UA patients were significantly increased compared with control subjects, but not in STEMI (Data not shown). The incongruous trend of miRNAs expression between UA and STEMI may reflect the complex process during progression of CAD. More importantly, these preliminary studies suggested that circulating miRNAs might be associated with subtype of coronary atherosclerotic pathology.

Numerous studies have shown that altered levels of circulating miRNAs in cardiovascular diseases. While the cardiovascular system is extremely sensitive to changes in miRNAs level and there is still a lack of consensus on the expression of miRNAs. The platelet-derived miR-17 was reported to predict adverse cardiovascular events in patients with CAD.^[39]^ However, a recent study did not found a prognostic value of miR-17 in patients with CAD, in contrast to miR-133a whose transcoronary concentration gradient did predict the cardiovascular outcome of these patients.^[40,41]^ Previous research demonstrates that miRNAs such as miR-126, miR-208, miR-223, and miR-499 were found to be consistently and significantly related to incident of CAD, which supporting our finding in the present study.^[37,42,43]^ Similarly, other putative miRNAs such as miR-133a, miR-221-3p, and miR-374-5p involved in response to AMI were suggested.^[44]^ However, these 3 miRNAs were moderately increased in our profile study. The high variability and poor overlap of the aberrant miRNAs expression profiles in difference study may be related to several vital influenced factors, including patient population, specimen source, study design, detection method, bioinformatics analysis method, as well as race and ethnicity. Furthermore, identification of novel miRNAs may become a key step in the future development of diagnostic biomarkers or therapeutic target for CAD, the biological roles of the other such as miR-340-3p, mir-125b-1-3p and miR-511-5p, which has no known association with cardiovascular disease, warrant further investigation.

Eight miRNAs were selected for RT-qPCR based on the RNA sequencing results. In generally, consistent results were obtained for all the miRNAs (Fig. 2). To evaluate the efficiency of these miRNAs for the diagnosis of CAD, ROC curves were constructed for up-regulated miRNA. MiR-142-3p showed the ability to distinguish UA from NCA efficiently, with an AUC value of 0.805 (95% CI: 0.672–0.938). In addition, miR-142-3p and miR-17-5p showed the ability to distinguish STEMI from NCA. Previously, both the miR-17 and miR-142 have been found to be dysregulated in (HF) and functional investigations have demonstrated serum extracellular vesicles promote proliferation of H9C2 cardiomyocytes by increasing miR-17-3p.^[45,46]^ We therefore speculate these altered miRNAs in the present study may thus be potential biomarkers for the detection and diagnosis of UA and STEMI, as well as other complications associated with CAD. However, the biological mechanisms of the dysregulation of these miRNAs and their therapeutic potential in CAD require further investigation.

It is well-known that microRNAs typically exert pivotal modulators effect on the expression of collections of messenger RNA targets that often have related functions, thereby governing complex biological processes.^[12,24]^ The potential biological function of miRNA specific target gene was also investigated in our study. Interestingly, these biological processes and KEGG pathway are closely related with the pathological process of CAD. According to our GO and KEGG pathway findings, most of these target genes were enriched in the biological process of regulation of cell communication, cellular localization, protein modification process, and regulation of transcription from RNA polymerase II promoter. Meanwhile, pathway significance concentration analysis revealed that several pathways were associated with inflammatory and immune processes, such as insulin signaling, hippo signaling pathway, and calcium signaling pathway. Previous publications reveal that marked alterations of miRNAs in circulation may reveal roles in pathologies of CAD as diverse as cell adhesion, endothelial cell proliferation, angiogenesis, oxidative stress, the inflammatory process, and metabolic disorders.^[47–49]^ Recent studies implicating the Hippo signaling pathway promotes cardiomyocyte proliferation by activating the insulin like growth factor.^[50]^ Similarly, Pi3kcb links Hippo-YAP and PI3K-AKT signaling pathways can promote cardiomyocyte proliferation and survival.^[51]^ These results demonstrate that the deregulated miRNAs in CAD play an important role in participating in many signaling pathways that control heart function. Still, there is a large part of studies regarding the roles of the pathways we identified in CAD that needs to be further explored.

Several limitations also need to be acknowledged in this study. Firstly, as a pilot study, our sampled only 18 individuals and thus differentially expressed miRNAs we found should be validated in studies with large samples. Secondly, as the findings of miRNAs are mainly based on bioinformatic observations in our research, their functional relevance should be validated in cell or animal models in future studies. Thirdly, evidence is accumulating that microvesicles represent major protective transport vehicles for miRNAs by separating them from circulating ribonuclease and microvesicles-packed miRNAs have been associated with cardiovascular diseases.^[46,52]^ In this study, however, we did not explore the miRNAs profiles derived from microvesicles-packed, while it is a topic which warrants further studies.

## 5 Conclusions

In summary, the results of this pilot study identified dysregulated miRNAs signature that can discriminate NCA from UA patients and STEMI patients. Importantly, these results provide new insights into the dynamic role of miRNAs expression associated with CAD pathogenesis and progression. Further mechanistic and clinical validation studies shall help to better understand their underlying clinical significance and role in the development of subtype of coronary atherosclerotic pathology.

## Acknowledgments

The authors would like to thank other colleagues whom were not listed in the authorship of Center for Cardiovascular Diseases, Clinical Core Laboratory and Center for Precision Medicine, Meizhou People's Hospital (Huangtang Hospital), Meizhou Hospital Affiliated to Sun Yat-sen University for their helpful comments on the manuscript.

## Author contributions

Pingsen Zhao conceived and designed the experiments; Zhixiong Zhong, Jingyuna Hou, and Pingsen Zhao recruited subjects and collected clinical data. Jingyuna Hou conducted the laboratory testing. Qifeng Zhang, Wei Zhong, Bin Li, Cunren Li, Zhidong Liu, and Min Yang helped to analyze the data. Pingsen Zhao, Zhixiong Zhong, and Jingyuna Hou prepare the manuscript. Pingsen Zhao and Zhixiong Zhong reviewed the manuscript.

**Conceptualization:** Pingsen Zhao.

**Data curation:** Zhixiong Zhong, Jingyuan Hou, Pingsen Zhao.

**Formal analysis:** Zhixiong Zhong, Jingyuan Hou, Pingsen Zhao.

**Funding acquisition:** Zhixiong Zhong, Pingsen Zhao.

**Investigation:** Pingsen Zhao.

**Methodology:** Zhixiong Zhong, Jingyuan Hou, Qifeng Zhang, Wei Zhong, Bin Li, Cunren Li, Zhidong Liu, Min Yang, Pingsen Zhao.

**Project administration:** Pingsen Zhao.

**Resources:** Zhixiong Zhong, Qifeng Zhang, Wei Zhong, Bin Li, Cunren Li, Zhidong Liu, Min Yang, Pingsen Zhao.

**Software:** Zhixiong Zhong, Jingyuan Hou, Qifeng Zhang, Pingsen Zhao.

**Supervision:** Pingsen Zhao.

**Validation:** Zhixiong Zhong, Jingyuan Hou, Wei Zhong, Pingsen Zhao.

**Visualization:** Pingsen Zhao.

**Writing – original draft:** Zhixiong Zhong, Jingyuan Hou, Pingsen Zhao.

**Writing – review and editing:** Pingsen Zhao.
